# Association Between Dietary Cholesterol Intake and Prediabetes: A Population‐Based Study

**DOI:** 10.1155/ije/8722312

**Published:** 2026-07-24

**Authors:** You-Fan Peng, Zihao Shi, Renjie Yin, Tian Xia, Qunyan Zhou, Jiayue Yang, Chunhua Wu

**Affiliations:** ^1^ Department of Respiratory and Critical Care Medicine, Affiliated Hospital of Youjiang Medical University for Nationalities, Baise, China, gxyyfy.cn; ^2^ Life Science and Clinical Medicine Research Center, Affiliated Hospital of Youjiang Medical University for Nationalities, Baise, China, gxyyfy.cn; ^3^ Department of Clinical Nutrition, Wuxi People’s Hospital, Wuxi Medical Center, The Affiliated Wuxi People’s Hospital of Nanjing Medical University, Wuxi, China, njmu.edu.cn; ^4^ Department of Endocrinology, Wuxi People’s Hospital, Wuxi Medical Center, The Affiliated Wuxi People’s Hospital of Nanjing Medical University, Wuxi, China, njmu.edu.cn

**Keywords:** dietary cholesterol intake, nutrients, prediabetes

## Abstract

**Background:**

Different dietary nutrients are associated with the risk of prediabetes. However, the association between dietary cholesterol intake and prediabetes is unclear. Thus, this study aimed to explore the association between dietary cholesterol intake and prediabetes.

**Methods:**

Data were extracted and analyzed from the National Health and Nutrition Examination Survey between 2011 and 2016. Self‐reported 24‐hour dietary recalls were used to evaluate dietary nutrient intake.

**Results:**

Participants with prediabetes exhibited a significantly higher dietary cholesterol intake compared with those with normoglycemia (241 [156, 367] vs. 263 [168, 381] mg, *p* = 0.003). Dietary cholesterol intake was significantly positively correlated with fasting blood glucose (*r* = 0.0607, *p* < 0.001) and hemoglobin A1c (*r* = 0.0486, *p* = 0.007) in all participants. Multivariable logistic regression analysis showed that higher dietary cholesterol intake was independently associated with prediabetes (OR = 1.241, 95% CI: 1.010–1.525, *p* = 0.040; OR = 1.288, 95% CI: 1.011–1.640, *p* = 0.040).

**Conclusion:**

Higher dietary cholesterol intake is independently associated with prediabetes, suggesting that dietary cholesterol intake restriction may be beneficial to prevent prediabetes.

## 1. Introduction

Prediabetes is a condition that elevated blood glucose levels below the threshold for a diagnosis of diabetes but associated with a higher risk of developing diabetes [1]. It has been well documented that prediabetes is associated with increased risk of diabetes [2]. Evidently, the prevention of prediabetes has positive significance for public health. It has been reported that decreasing meat and seafood consumption and increasing intakes of eggs, coffee, and milk may be associated with a lower risk of prediabetes [3]. Increased total dietary fiber intake from fruit sources has been associated with a lower risk of prediabetes [4]. Higher dietary acid load and animal protein intake have been associated with an increased risk of prediabetes [5, 6]. However, as far as we know, the association has not been revealed between dietary cholesterol intake and prediabetes. Thus, the aim was to examine whether dietary cholesterol intake was associated with prediabetes in this study.

## 2. Methods

### 2.1. Study Population

A total of 1743 prediabetes and 1339 normoglycemia participants were included from the National Health and Nutrition Examination Survey between 2011 and 2016. Diagnosis of prediabetes was based on the American Diabetes Association criteria [7]. This study excluded the following participants: (1) diabetes, pregnancy, or unusual diet; (2) those aged < 18 years; (3) dietary supplement use; and (4) participants with missing data for the study variables, except for dietary intake assessed during the second 24‐h dietary recall interviews. This study was approved by the National Center for Health Statistics Research Ethics Review Board (Protocol #2011–17) and adhered the principles of the Declaration of Helsinki. All participants provided written informed consent.

### 2.2. Data Collection

Age and gender were obtained from demographic variables. Smoking status, alcohol consumption, family history of diabetes, and physical activity data were collected from questionnaire data. Body mass index (BMI) data were collected from examination data. Fasting blood samples were used for laboratory examinations including fasting blood glucose (FBG), 2‐hour postprandial blood glucose (2h‐PBG), hemoglobin A1c (HbA1c), total cholesterol (TC), high‐density lipoprotein cholesterol (HDL‐C), low‐density lipoprotein cholesterol (LDL‐C), and triglycerides (TG).

A 24‐hour dietary recall interview was used to assess participants’ daily dietary intake. The first set of data was collected on‐site at a mobile inspection center, and subsequently, the second set was collected over the phone 3–10 days later. Daily dietary intake was estimated by two 24‐hour dietary recalls. All participants completed the first recall. For those who completed both recalls, daily intake was calculated as the mean of the two; for those who completed only the first recall, the single recall value was used.

### 2.3. Statistical Analysis

Categorical variables are expressed as frequency (percentage), and non‐normally distributed continuous variables are expressed as median (interquartile range). The chi‐square test was used to compare the differences in categorical variables, and the Mann–Whitney *U* test was used to compare the differences in continuous variables. The correlation between dietary cholesterol intake and continuous variables was assessed with Spearman’s rank correlation coefficient. Univariable and multivariable logistic regression analyses were used to calculate the odds ratio (OR) with 95% confidence interval (CI) for risk of prediabetes. Variables that were considered clinically relevant or that showed a significant association in univariable logistic regression analysis were entered into multivariable logistic regression analysis. A restricted cubic spline plot was used to exhibit the association between dietary cholesterol intake and prediabetes with adjustment for gender, age, BMI, TC, TG, HDL‐C to LDL‐C ratio, smoking status, drinking, close relatives with diabetes, sedentary activity, hypertension history, dietary carbohydrate intake, dietary protein intake, and dietary total fat intake. All statistic assessments were two‐sided, and a *p* value < 0.05 was considered as significant. The statistical analyses were performed by Stata Statistical Software Version 15.0 (Stata Corporation, College Station, TX, USA).

## 3. Results

### 3.1. Comparative Characteristics of Participants With Prediabetes and Those With Normoglycemia

The baseline characteristics of the participants with prediabetes and those with normoglycemia are shown in Table 1. Dietary cholesterol intake was significantly higher in participants with prediabetes than in those with normoglycemia (263 [168, 381] vs. 241 [156, 367] mg, *p* = 0.003). There were significantly differences in age, gender, BMI, TC, TG, HDL‐C to LDL‐C ratio, drinking, smoking status, close relative with diabetes, and hypertension history between participants with prediabetes and those with normoglycemia (all *p* < 0.05). However, no significant differences were observed between participants with prediabetes and those with normoglycemia in sedentary activity or in dietary intake of carbohydrates, protein, and total fat (all *p* > 0.05).

**TABLE 1:** The characteristics of the participants with prediabetes and with those normoglycemia.

Variables	Normoglycemia	Prediabetes	*p* value
	*N* = 1339	*N* = 1743	
Age (years)	37 (27, 51)	53 (38, 65)	< 0.001
Males, *n* (%)	591 (44.14)	997 (57.20)	< 0.001
BMI (kg/m^2^)	25.9 (22.7, 29.6)	28.4 (24.7, 32.4)	< 0.001
TC (mg/dL)	184 (162, 210)	195 (170, 221)	< 0.001
TG (mg/dL)	85 (61, 122)	107 (75, 158)	< 0.001
HDL‐C to LDL‐C ratio	0.512 (0.386, 0.689)	0.450 (0.342, 0.600)	< 0.001
Drinking, *n* (%)	1035 (77.30)	1269 (72.81)	0.004
Smoking status, *n* (%)			
Never smoker	833 (62.21)	948 (54.39)	< 0.001
Ever smoker	256 (19.12)	472 (27.08)	
Current smoker	250 (18.67)	323 (18.53)	
Close relatives with diabetes, *n* (%)	432 (32.36)	642 (36.83)	0.008
Sedentary activity (minutes)	360 (240, 480)	360 (240, 480)	0.229
Hypertension history, *n* (%)	261 (19.49)	653 (37.46)	< 0.001
Dietary nutrient intake			
Carbohydrate (gm)	240.27 (182.50, 310.39)	243.14 (182.99, 313.44)	0.625
Protein (gm)	77.57 (60.57, 100.24)	79.62 (59.47, 102.01)	0.613
Total fat (gm)	72.54 (53.74, 98.25)	74.81 (54.80, 100.85)	0.232
Cholesterol (mg)	241 (156, 367)	263 (168, 381)	0.003

*Note:* TG, triglycerides.

Abbreviations: BMI, body mass index; HDL‐C, high‐density lipoprotein cholesterol; LDL‐C, low‐density lipoprotein cholesterol; TC, total cholesterol.

### 3.2. Dietary Cholesterol Intake and Metabolic Parameters

Dietary cholesterol intake was significantly positively correlated with BMI (*r* = 0.1012, *p* < 0.001), FBG (*r* = 0.0607, *p* < 0.001), HbA1c (*r* = 0.0486, *p* = 0.007), and dietary intake of carbohydrate (*r* = 0.2554, *p* < 0.001), protein (*r* = 0.6512, *p* < 0.001), and total fat (*r* = 0.5523, *p* < 0.001) and was significantly negatively correlated with age (*r* = −0.0661, *p* < 0.001) and HDL‐C to LDL‐C ratio (*r* = −0.0834, *p* < 0.001) in all participants. However, dietary cholesterol intake was not significantly correlated with 2h‐PBG, TC, and TG in all participants (all *p* > 0.05).

### 3.3. Univariable Analysis Between Dietary Cholesterol Intake and Prediabetes

In univariable logistic regression analysis, male gender (OR = 1.691, 95% CI: 1.465 to 1.953, *p* < 0.001), older age (OR = 1.042, 95% CI: 1.037 to 1.047, *p* < 0.001), higher BMI (OR = 1.073, 95% CI: 1.059 to 1.087, *p* < 0.001), higher TC (OR = 1.006, 95% CI: 1.004 to 1.008, *p* < 0.001), higher TG (OR = 1.006, 95% CI:1.005 to 1.007, *p* < 0.001), lower HDL‐C to LDL‐C ratio (OR = 0.333, 95% CI: 0.248 to 0.448, *p* < 0.001), ever smoking (OR = 1.620, 95% CI: 1.355 to 1.936, *p* < 0.001), nondrinking (OR = 0.786, 95% CI: 0.666 to 0.928, *p* = 0.004), close relative with diabetes (OR = 1.224, 95% CI:1.053 to 1.423, *p* = 0.008), hypertension history (OR = 2.274, 95% CI: 2.095 to 2.922, *p* < 0.001), and higher dietary cholesterol intake (OR = 1.199, 95% CI: 1.008–1.428, *p* = 0.041; OR = 1.287, 95% CI: 1.081–1.532, *p* = 0.005) were significantly associated with prediabetes, as shown in Table 2.

**TABLE 2:** Relative odds of prediabetes according to univariable and multivariable analyses in participants.

Variables	Univariable analysis	*p* value	Multivariable analysis	*p* value
	OR (95%CI)		OR (95%CI)	
Males	1.691 (1.465–1.953)	< 0.001	1.995 (1.660–2.398)	< 0.001
Age	1.042 (1.037–1.047)	< 0.001	1.040 (1.034–1.046)	< 0.001
BMI	1.073 (1.059–1.087)	< 0.001	1.066 (1.051–1.082)	< 0.001
TC	1.006 (1.004–1.008)	< 0.001	1.002 (1.000–1.005)	0.053
TG	1.006 (1.005–1.007)	< 0.001	1.003 (1.001–1.004)	< 0.001
HDL‐C to LDL‐C ratio	0.333 (0.248–0.448)	< 0.001	0.896 (0.612–1.312)	0.574
Smoking status				
Never smoker	1 (Reference)		1 (Reference)	
Ever smoker	1.620 (1.355–1.936)	< 0.001	1.036 (0.840–1.277)	0.741
Current smoker	1.135 (0.939–1.372)	0.190	1.132 (0.911–1.406)	0.263
Drinking	0.786 (0.666–0.928)	0.004	0.704 (0.577–0.859)	0.001
Close relatives with diabetes	1.224 (1.053–1.423)	0.008	1.191 (1.007–1.409)	0.042
Sedentary activity	1.000 (1.000–1.000)	0.306	1.000 (1.000–1.000)	0.111
Hypertension history	2.274 (2.095–2.922)	< 0.001	1.172 (0.963–1.425)	0.113
Dietary nutrient intake				
Carbohydrate	1.000 (0.999–1.001)	0.859	1.001 (0.999–1.002)	0.297
Protein	1.000 (0.998–1.002)	0.733	0.998 (0.995–1.002)	0.373
Total fat	1.000 (0.998–1.002)	0.738	0.999 (0.996–1.002)	0.564
Cholesterol (tertile)				
T1	1 (reference)		1 (reference)	
T2	1.199 (1.008–1.428)	0.041	1.241 (1.010–1.525)	0.040
T3	1.287 (1.081–1.532)	0.005	1.288 (1.011–1.640)	0.040

*Note*: TG, triglycerides.

Abbreviations: BMI, body mass index; HDL‐C, high‐density lipoprotein cholesterol; LDL‐C, low‐density lipoprotein cholesterol; TC, total cholesterol.

### 3.4. Multivariable Analysis Between Dietary Cholesterol Intake and Prediabetes

**FIGURE 1:**
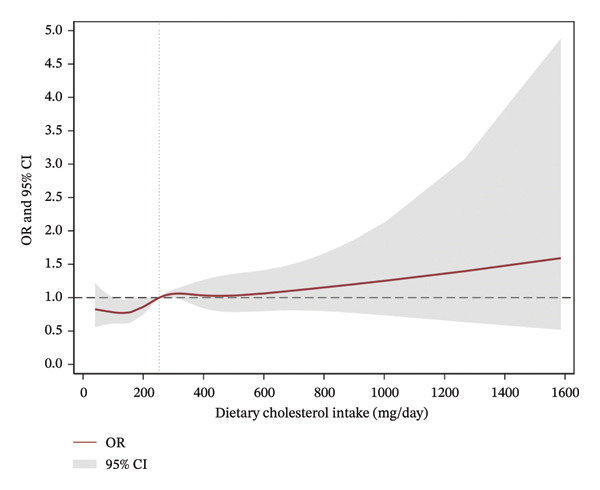
Restricted cubic spline plot between dietary cholesterol intake and prediabetes.

In multivariable logistic regression analysis, after gender, age, BMI, TC, TG, HDL‐C to LDL‐C ratio, smoking status, drinking, close relative with diabetes, sedentary activity, hypertension history, and dietary intake of carbohydrate, protein, and total fat were adjusted, the results showed that higher dietary cholesterol intake was independently associated with prediabetes (OR = 1.241, 95% CI: 1.010–1.525, *p* = 0.040; OR = 1.288, 95% CI: 1.011–1.640, *p* = 0.040), as shown in Figure 1, and that male gender (OR = 1.995, 95% CI: 1.660 to 2.398, *p* < 0.001), older age (OR = 1.040, 95% CI: 1.034 to 1.046, *p* < 0.001), higher BMI (OR = 1.066, 95% CI: 1.051 to 1.082, *p* < 0.001), higher TG (OR = 1.003, 95% CI: 1.001 to 1.004, *p* < 0.001), nondrinking (OR = 0.704, 95% CI: 0.577 to 0.859, *p* = 0.001), and close relatives with diabetes (OR = 1.191, 95% CI: 1.007 to 1.409, *p* = 0.042) were also independently associated with prediabetes, as shown in Table 2.

## 4. Discussion

Cholesterol is one of the most common nutrients, and it has a significant function in the human body [8]. Reducing excessive dietary cholesterol intake has been regarded as a way to attenuate obesity‐associated metabolic diseases [9]. It has been reported that the control of dietary cholesterol intake may help improve nonalcoholic fatty liver disease [10]. An association has been suggested between increased dietary cholesterol intake and a higher risk of metabolic syndrome [11]. Increased dietary cholesterol intake has been associated with a risk of gestational diabetes mellitus [12]. Interestingly, a meta‐analysis revealed a positive dose–response association between dietary cholesterol intake and the incidence of Type 2 diabetes mellitus [13]. Our study found that dietary cholesterol intake was higher in participants with prediabetes than in those with those normoglycemia, and that higher dietary cholesterol intake maintained an independent association with prediabetes.

In the present study, dietary cholesterol intake was significantly positively correlated with FBG and HbA1c, suggesting that increased dietary cholesterol intake may impair the regulation of blood glucose. Indeed, the accumulation of cholesterol may damage the function of pancreatic beta cells [14]. Higher levels of cholesterol in the islets suppress ATP‐binding cassette transporter A1 expression, suggesting that the abnormalities of cholesterol metabolism may result in impaired *β*‐cell function in diabetes [15]. Increased cholesterol intake induces the accumulation of hepatic cholesterol esters and TG, activating nuclear transcription factor‐liver *X* receptors (LXR) [16]. However, LXR activation contributes to the dysregulation of hepatic insulin signaling and exacerbation of insulin resistance by reducing insulin receptor beta subunit mass [17]. In addition, dietary cholesterol intake exacerbates macrophage accumulation and local inflammation in adipose tissue, which is associated with insulin resistance [18].

We observed that the difference in dietary cholesterol intake between participants with prediabetes and those with normoglycemia was small. Considering the long‐term nature of dietary exposure, the subtle differences such as dietary cholesterol intake also may play a role in the risk of prediabetes. On the other hand, the current findings suggested that even a small increase in dietary cholesterol intake was independently associated with prediabetes. The result underscores the significant impact of dietary cholesterol intake on prediabetes risk, and the strict control of dietary cholesterol intake may be even more necessary to reduce the risk of prediabetes.

There are several limitations in this study. First, we cannot infer a causal association between dietary cholesterol intake and the risk of prediabetes due to the cross‐sectional design of this study. Therefore, it remains unclear whether increased dietary cholesterol intake increases the risk of prediabetes, or participants with prediabetes are more inclined to consume cholesterol‐rich foods during disease status. Obviously, prospective cohort studies are needed to confirm the causality. Second, the assessment of dietary nutrient intake main includes 24‐hour dietary recalls, food frequency questionnaires, and food records. Despite the 24‐hour dietary recalls are less biased for the assessment of dietary nutrient intake than food frequency questionnaires and food records [19]. This study only relied on self‐reported 24‐hour recalls to evaluate dietary nutrient intake. However, the solely self‐reported 24‐hour recalls for estimation of dietary nutrient intake may still lead to potential bias, which may affect the current conclusions. Therefore, multiple approaches to assess dietary nutrient intake should be further considered in future studies. Third, other potential factors related to prediabetes, such as genetic predisposition, medication use, and lifestyle, were not adjusted in the multivariate analysis. Fourth, the current study did not assess an association between dietary cholesterol intake and islet cell function, especially in participants with prediabetes.

In conclusion, the study observed an independent association between increased dietary cholesterol intake and prediabetes. Consequently, dietary cholesterol intake control may be beneficial in preventing prediabetes. However, further studies are needed to confirm the current findings.

## Author Contributions

Chunhua Wu, Jiayue Yang, and You‐Fan Peng conceived and designed the study. Chunhua Wu, Tian Xia, Qunyan Zhou, and Renjie Yin extracted the data. Chunhua Wu, You‐Fan Peng, and Zihao Shi analyzed the data. You‐Fan Peng and Zihao Shi draft the manuscript. You‐Fan Peng and Chunhua Wu revised the manuscript.

## Funding

The authors have nothing to report.

## Disclosure

All authors have approved the final version of the manuscript.

## Conflicts of Interest

The authors declare no conflicts of interest.

## Data Availability

The data used in this study are publicly available from the National Health and Nutrition Examination Survey (NHANES) at https://www.cdc.gov/nchs/nhanes/.
